# Novel Recombinant Sapovirus

**DOI:** 10.3201/eid1010.040395

**Published:** 2004-10

**Authors:** Kazuhiko Katayama, Tatsuya Miyoshi, Kiyoko Uchino, Tomoichiro Oka, Tomoyuki Tanaka, Naokazu Takeda, Grant S. Hansman

**Affiliations:** * National Institute of Infectious Diseases, Tokyo, Japan;; † Sakai City Institute of Public Health, Sakai, Japan; and; ‡ University of Tokyo, Tokyo, Japan

**Keywords:** Keywords: dispatch, recombination, Norovirus, Sapovirus, genetic analysis, polymerase, capsid

## Abstract

We determined the complete genome sequences of two sapovirus strains isolated in Thailand and Japan. One of these strains represented a novel, naturally occurring recombinant sapovirus. Evidence suggested the recombination site was at the polymerase-capsid junction within open reading frame one.

The positive-sense polyadenylated single-stranded RNA virus family *Caliciviridae* contains four genera, *Norovirus*, *Sapovirus*, *Lagovirus*, and *Vesivirus* (*1*). Human norovirus is the most important cause of outbreaks of gastroenteritis in the United States and infects all age groups (*2*). Human sapovirus is also a causative agent of gastroenteritis but is more frequent in young children than in adults (*3*). Most animal caliciviruses are grouped within the other two genera. In 1999, Jiang et al. (*4*) identified the first naturally occurring human recombinant norovirus, and several other strains were later described as recombinants (*5**–**8*). Evidence suggested that the recombination event occurred at the junction of open reading frames one and two (ORF1 and ORF2), but this finding was not proven. Norovirus ORF1 encodes nonstructural proteins, including the RNA-dependent RNA polymerase, ORF2 encodes the capsid protein, and ORF3 encodes a small capsid protein (*1*). Nucleotide sequence of the polymerase and capsid junction generally is conserved among the human norovirus genotypes (*4**,**6*), which likely facilitates a recombination event when nucleic acid sequences of parental strains come into physical contact in infected cells, e.g., during copy choice recombination (*9*).

## The Study

We used genetic analysis to investigate a novel, naturally occurring recombinant sapovirus. Two strains were used for the analysis, Mc10 strain (GenBank accession no. AY237420), isolated from an infant hospitalized with acute gastroenteritis in Chiang Mai, Thailand, in 2000 (*5*), and C12 strain (AY603425), isolated from an infant with gastroenteritis in Sakai, Japan, in 2001 (unpub. data). Although the original polymerase chain reaction (PCR) primer sets that detected these two strains were different, both were directed toward the conserved 5´ end of the capsid gene and have been shown to detect a broad range of sapovirus sequences in genogroup I (GI) and GII (*5**,**10*). For Mc10, primers SV5317 and SV5749 were used; for C12, primers SV-F11 and SV-R1 were used.

**Figure 1:**
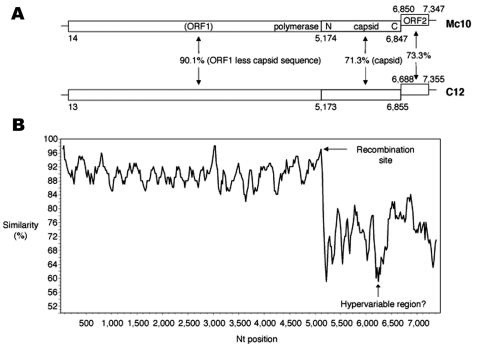
A) The genomic organization of Mc10 and C12 strains. B) the SimPlot analysis of Mc10 and C12. Mc10 genome sequence was compared to C12 by using a window size of 100 bp with an increment of 20 bp. All gaps were removed. The recombination site is suspected to be located between polymerase and capsid genes, as shown by the arrow. The possible hypervariable region for the capsid protein is also shown.

The complete genomes for Mc10 and C12 were determined as previously described (*6*). As shown in Figure 1A, the sapovirus genome has an organization slightly different from that of the norovirus genome. ORF1 encodes nonstructural proteins, polymerase, and the capsid protein, and ORF2 encodes a small protein (*1*).

**Figure 2:**
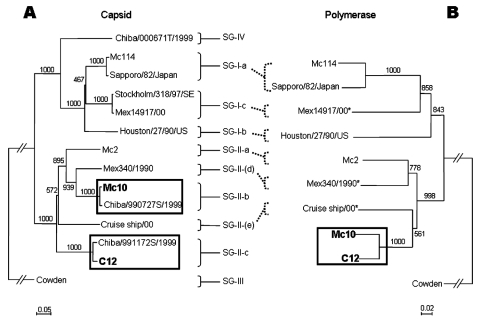
Phylogenetic analysis of (A) capsid (376 nt) and (B) polymerase (289 nt) sequences of Mc10, C12, and additional strains in GenBank. Sapovirus capsid sequences were classified on the basis of the scheme of Okada et al. (*10*). Two unclassified strains, Mex340/1990 and Cruise ship/00, were assigned SG-II-(d) and SG-II-(e). The asterisks indicate noncontinuous polymerase-capsid sequences. The numbers on each branch indicate the bootstrap values for the genotype. Bootstrap values of >950 were considered statistically significant for the grouping (*6*). The scale represents nucleotide substitutions per site. GenBank accession no. for the reference strains are as follows: Chiba/000671T/1999, AJ412805; Chiba/990727S/1999, AJ412795; Chiba/991172S/1999, AJ412797; Mc114, AY237422; Cruise ship/00, AY289804 and AY157863; Cowden, AF182760; Houston/27/90/US, U95644; Mc2, AY237419; Mc10, AY237420; Mex340/1990, AF435809 and AF435812; Mex14917/00, AF435813 and AF35810; Sapporo/82/Japan, U65427; and Stockholm/318/97/SE, AF194182.

Initially, we grouped Mc10 and C12 into two distinct GII clusters (i.e., genotypes), on the basis of their capsid sequences (Figure 2A) and the phylogenetic classification scheme of Okada et al. (*10*). In addition, the overall genomic nucleotide similarity between Mc10 and C12 was 84.3%, while ORF1 and ORF2 shared 85.5% and 73.3% nucleotide identity, respectively. These results corresponded with the capsid-based grouping shown in Figure 2A. By comparing sequence similarity across the length of the genomes with SimPlot with a window size of 100 (*11*), we discovered a potential recombination site, where the similarity analysis showed a sudden drop in nucleotide identity after the polymerase region (Figure 1B). Nucleotide sequence analysis of ORF1 less the capsid sequence and the capsid sequence indicated 90.1% and 71.3% nucleotide identity, respectively (Figure 1A). To additionally illustrate the nucleotide identities of ORF1 less the capsid sequence, a phylogenetic tree of polymerase sequences of Mc10, C12, and other available strains was developed (Figure 2B). However, for three strains (Mex14917/00, Mex340/1990, and Cruise ship/00), the polymerase and capsid sequences of ORF1 were not continuous, i.e., they may represent two different strains. Nevertheless, Mc10 and C12 were in the same cluster by polymerase-based grouping but were in distinct clusters by capsid-based grouping (Figure 2). All other strains maintained clusters by polymerase- and capsid-based groupings.

These findings showed Mc10 and C12 had high sequence identity up to the beginning of the capsid region where the sequence identity was considerably lower. These results are easily explained by a recombination event, a single point recombination event occurring at the polymerase-capsid junction. At the end of the polymerase region, there were 44 nt, which included the first 8 nt of the capsid gene and showed 100% homology. After these nucleotides, the identity decreased and was clearly different, as shown in Figure 1B. This conserved region may represent the break and rejoin site for Mc10 and C12 during viral replication, although direct evidence for this event is lacking.

A sudden drop was indicated, followed by a rise in nucleotide identity between nt 6,250 and 6,500 (Figure 1B). Although our initial hypothesis was that another recombination event occurred, closer inspection indicated that this region corresponded to amino acids 358 and 440 for the capsid protein and likely represented the hypervariable region, as described recently in the structural analysis of sapovirus capsid protein (*12*). For recombinant norovirus strains, we also observed a sudden decrease in nucleotide identity in the related capsid region (*13*), which represents the outermost protruding domain (P2) and is subject to immune pressure (*14*). For these reasons, a low homology, even between closely related strains, is generally seen in this region (*6*), although further studies by sequence analysis with other strains are needed.

In a recent study, we genetically and antigenically analyzed two recombinant norovirus strains (*13*). When the polymerase-based grouping was performed, these two strains clustered together; when capsid-based grouping was performed, these two strains belonged in two distinct genotypes. When we compared the cross-reactivity of these two viruslike particles (VLPs) and hyperimmune sera against the VLPs, we found distinct antigenic types for the VLPs, although a considerable level of cross-reactivity was found between them. We recently expressed C12 capsid protein that resulted in the formation of VLPs, but we were unsuccessful in expressing Mc10 VLPs (G.S. Hansman, unpub. data); therefore the antigenicity of these two strains remains unknown.

Jiang et al. (*4*) reported two potential parental norovirus strains that were cocirculating in the same geographic region (Mendoza, Argentina, in 1995), which provides some evidence for where and when the recombination event may have occurred. In addition, Jiang identified the progeny strain from the event, the Arg320 strain. In our study, Mc10 and C12 were isolated from Thailand and Japan, respectively, but we have no evidence for the place and time of the event. While the genetic analysis for Mc10 and C12 identified a possible recombinant sapovirus strain, the analysis does not clarify which of the two strains was the parent strain and which was the progeny strain. Further extensive studies are needed that perform sequence analysis of polymerase and capsid genes and compare results with analysis of other strains. Nevertheless, other strains with capsid sequences that closely match those of Mc10 and C12 are in the public database, which suggests the circulation of other recombinant sapovirus strains.

## Conclusions

Recombination and evolution are important survival events for all living creatures as well as viruses. These events in viruses are not completely understood, but they can be potentially dangerous for host species, and they likely influence vaccine designs (*15*). From our studies, the human sapovirus and norovirus recombination appears limited to the intragenogroup because no intergenogroup or intergenus recombination has yet been identified and recombination only occurs at the polymerase-capsid junction. Finally, the results of this study have increased our awareness of recombination in the *Sapovirus* genus and may have an influence on the future phylogenetic classification of sapovirus strains.
